# Antiplasmodial activity, structure–activity relationship and studies on the action of novel benzimidazole derivatives

**DOI:** 10.1038/s41598-022-27351-z

**Published:** 2023-01-06

**Authors:** Nerea Escala, Laura M. Pineda, Michelle G. Ng, Lorena M. Coronado, Carmenza Spadafora, Esther del Olmo

**Affiliations:** 1 Departamento de Ciencias Farmacéuticas: Química Farmacéutica, Facultad de Farmacia, Universidad de Salamanca, CIETUS, IBSAL, 37007 Salamanca, Spain; 2 Center of Cellular and Molecular Biology of Diseases, Instituto de Investigaciones Científicas y Servicios de Alta Tecnología, City of Knowledge, Clayton, Apartado 0816-02852, Panama City, Panama

**Keywords:** Chemical biology, Drug discovery

## Abstract

Malaria cases and deaths keep being excessively high every year. Some inroads gained in the last two decades have been eroded especially due to the surge of resistance to most antimalarials. The search for new molecules that can replace the ones currently in use cannot stop. In this report, the synthesis of benzimidazole derivatives guided by structure–activity parameters is presented. Thirty-six molecules obtained are analyzed according to their activity against *P. falciparum* HB3 strain based on the type of substituent on rings A and B, their electron donor/withdrawing, as well as their dimension/spatial properties. There is a preference for electron donating groups on ring A, such as Me in position 5, or better, 5, 6-diMe. Ring B must be of the pyridine type such as picolinamide, other modifications are generally not favorable. Two molecules, **1** and **33** displayed antiplasmodial activity in the high nanomolar range against the chloroquine sensitive strain, with selectivity indexes above 10. Activity results of **1**, **12** and **16** on a chloroquine resistance strain indicated an activity close to chloroquine for compound **1**. Analysis of some of their effect on the parasites seem to suggest that **1** and **33** affect only the parasite and use a route other than interference with hemozoin biocrystallization, the route used by chloroquine and most antimalarials.

## Introduction

Malaria is a potentially mortal febrile disease that is caused by protozoa of the genus *Plasmodium*. The parasite is transmitted by the bite of an infected female *Anopheles* mosquito^1^. Malaria is a preventable and treatable disease; however, it continues to be a global health problem, and according to the World Health Organization (WHO) 2021 report^2^, 241 million cases were reported in 2020, being the African continent the region with the highest global malaria burden^2^. Since 2021, the WHO recommends the use of the RTS,S/AS01 vaccine (Mosquirix™) among children living in regions with moderate to high *Plasmodium falciparum* (*Pf*) transmission^3^, although the efficacy of this vaccine is of only 28.3% in approximately 4 years^4^. The best existing treatment is the artemisinin-based combination therapy (ACT), artemether–lumefantrine, artesunate–amodiaquine, artesunate–mefloquine, artesunate–sulfadoxine–pyrimethamine, and dihydroartemisinin–piperaquine. A sixth ACT (artesunate–pyronaridine) has recently been included^5^. However, in the last year’s antimalarial drug resistances to ACT have appeared, with a mutation in the Kelch13 gene strongly related to artemisinin resistances, and the Greater Mekong subregion is the most affected, presenting a new threat for the management of malaria^6^. Recently, the same resistance has also been identified in Rwanda, which to date had not been reported in the African continent^7^. The need for new antimalarials is a pressing task.

The benzimidazole skeleton (BZ) plays an important role in the medicinal chemistry field. Substitutions in different positions of the molecule provide compounds with a wide variety of applications such as antihypertensive^8^, antidiabetic^9^, antitumoral^10^, antimicrobial^11^ or anti-inflammatory^12^, among others.

In the last 5 years several research groups have worked on the synthesis of BZ derivatives with potential antimalarial activity. On the one hand, 2-phenyl-1*H*-benzimidazole derivatives exhibited a good activity in vitro against *P. falciparum* with IC_50_ values of the most potent compounds ranging from 18 nM to 1.30 µM^13–16^. The inclusion of a spacer between the BZ nucleus and the aromatic fragment in C-2 give the molecules the possibility to adopt different conformations. In this way, Attram et al. included an amino group in C-2, obtaining in vitro fast-acting anti-*Plasmodium* compounds with a hit compound which showed an IC_50_ of 79 nM against the chloroquine-sensitive strain NF54 and 335 nM against the multi-resistant strain K1^17^. The introduction of an acrylonitrile group as a linker also showed good *Pf* inhibitory activity of BZ derivatives, with an IC_50_ value for the best compound of 0.69 and 3.41 μM in 3D7 and RKL9 strains, respectively^18^.

Substitutions in the *N*-1 position of BZ provides good antiplasmodial derivatives. Thus, some research groups have synthetized *N*-1 aryl, hetaryl or alkyl derivatives obtaining interesting results, with activities ranging from 6.4 nM to 1.5 μM for most active compounds^19–23^.

Tricyclic derivatives such as pyrido[1,2-*a*]benzimidazole seem to be a good alternative for malaria treatment, providing compounds with submicromolar IC_50_ values, and reductions of parasitemia above 95%^24–28^. The bioactivity of Schiff bases attached to BZ nucleus is described in previous literature; based on it, the antimalarial activity of some novel derivatives has been evaluated recently, exhibiting favorable results in vitro^29^ and with some derivatives displaying IC_50_ values ranging from 26.9 to 28.3 µg/mL. More recently, Aragon et al.^30^ described the antiplasmodial activity of some metal-based Schiff bases-BZ with EC_50_ values ranging from 12.8 to 18.3 µg/mL.

Furthermore, the obtaining of derivatives related to the antiallergic drug astemizole^31–33^ or lerisetron^34^, showed congeners with good IC_50_ values, from 47 nM to 1.7 μM against NF54 and K1 strains; moreover, derivatives related to some natural products, as carvacrol, showed activity values of 0.48 – 1.76 μg/mL^35^. Ferroquine, a ferrocenyl-chloroquine in phase 2b studies, led to Baartzes et al. to the obtaining and evaluation of ferrocenyl and neutral and cationic iridium (III) and rhodium (III) aminoquinoline-benzimidazole hybrids^36,37^ and 2(2-pyridyl)benzimidazole^38^. In the same sense, iridium(III), rhodium(III) and ruthenium (II) BZ-complexes were also tested against *Pf*^39^. Organometallic-BZ complexes exhibited IC_50_ values in the low submicromolar range (0.12–5.17 μM).

Bearing these facts in mind, in the present study we explore the in vitro activity of some BZ derivatives against *Pf*. These compounds bear an amide spacer between the BZ nucleus and the heterocyclic fragment (ring B). The amide fragment allows the molecules the possibility of adopting an exocyclic tautomerization, which could contribute to establish an additional interaction with its target and perhaps increase the activity of the compounds. The introduction of electron donor or acceptor groups on ring A would allow, on the one hand, to enhance (or not) the formation of the exocyclic tautomer and, on the other hand, to increase the BZ size to fit better with the target, or establish an additional bond as could happen with the methoxy group (an additional hydrogen bond could be established) thus increasing the activity. Regarding ring B, the convenience of medium-sized (pyridine) or larger (quinoline, isoquinoline) heterocycles has been explored. Even the presence of certain substituents (type and position) on the pyridine ring. Additionally, the cytotoxicity against the Vero cell line for most active compounds is described, and the corresponding selectivity index (SI) calculated. A set of mechanistic studies were included for some compounds with good activities but inadequate selectivity and they were compared in head-to-head experiments with the lead compounds BZ **1** and **33** to obtain clues on the possible differentiation between them in terms of their action on the parasites.

## Results and discussion

### Chemical synthesis

**Figure 1:**
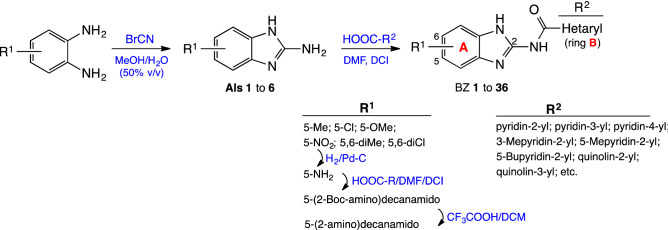
Procedure for the synthesis of 2-amidobenzimidazole derivatives.

To obtain the 2-arylcarbamidoBZ derivatives, the corresponding 2-amino-1*H*-benzimidazole intermediate (**AI**) should be previously synthesized. The procedure to obtain **AI 1–5** is indicated in a Escala et al. previous work^40^. Here we indicate as an example compound **AI-6** obtaining, solutions of the 4-nitro-1,2-phenylendiamine and cyanogen bromide in aqueous methanol (50% v/v) were prepared separately. Then, the solutions were mixed and maintained with continuous magnetic stirring at room temperature for 48 h^41^, to provide **AI-6** in 95% yield. The coupling of intermediates **AI-1**/**AI-6** with different acids in the presence of 1,1′-carbonyldiimidazole (DCI) as catalyst at room temperature for 16 h, allowed us to obtain BZ **1** to **29** and BZ **33** to **36** with yields ranging from 27 to 69%, (Fig. 1).

BZ **30** was obtained by the reduction of **29** under H_2_ atmosphere with a yield of 85%. BZ **31** was synthetized by coupling **30** with 2-(Boc-amino)decanoic acid and DCI in 32% yield. The procedure for obtaining 2-(Boc-amino)decanoic acid is indicated in Valderas et al^42^. Boc deprotection of **31** with trifluoroacetic acid provided the corresponding free amino derivative **32** in 76%.

All the compounds obtained (intermediate and final) were properly characterized according to their physicochemical properties. For experimental details, description of synthesized intermediates and characterization of the most active final compounds see the Experimental: Chemistry section; for the rest of the compounds see Supplementary Information.

### Antiplasmodial activity

In vitro *activity of BZ.* All BZ derivatives were tested at 10 μM against the HB3 strain of *Pf*, and for those compounds with a percentage of growth inhibition (% GI) higher than 50%, the IC_50_ values were determined. In addition, their cytotoxicity on epithelial Vero cell cultures was evaluated to calculate their selectivity indexes (SI), Table 1. An index of 10 or above is expected for a lead compound.

**Table 1:** Growth inhibition, IC_50_ cytotoxicity and selectivity index of 2-amidobenzimidazole derivatives in HB3 *P. falciparum* parasites.

N°	R^1^	R^2^	GI^a^ (%)	IC_50_ (μM)	Cytotoxicity^b^ CC_50_ (μM)	SI
1	5-CH_3_	pyridin-2-yl	**93.5**	**0.98**	**14.8**	**14.5**
2	5-CH_3_	pyridin-3-yl	0.0	nc	nc	nc
3	5-CH_3_	pyridin-4-yl	0.0	nc	nc	nc
4	5-CH_3_	3-methylpyridin-2-yl	92.4	2.5	6.8	2.8
5	5-CH_3_	5-methylpyridin-2-yl	100.0	1.5	4.5	1.6
6	5-CH_3_	5-butylpyridin-2-yl	90.9	2.4	6.6	2.7
7	5-CH_3_	5-phenylpyridin-2-yl	57.5	7.8	19.5	2.5
8	5-CH_3_	5-cyanopyridin-2-yl	37.4	nc	nc	nc
9	5-CH_3_	quinolin-2-yl	73.1	14.1	63.0	4.5
10	5-CH_3_	quinolin-4-yl	60.5	nc	nc	nc
11	5-CH_3_	isoquinolin-1-yl	0.0	nc	nc	nc
12	5-Cl	pyridin-2-yl	91.4	1.8	15.4	8.7
13	5-Cl	3-methylpyridin-2-yl	87.3	2.8	8.6	3.0
14	5-Cl	3-chloropyridin-2-yl	11.9	nc	nc	nc
15	5-Cl	4-methoxypyridin-2-yl	89.8	1.5	2.0	1.4
16	5-Cl	5-methylpyridin-2-yl	85.6	2.4	7.2	3.0
17	5-Cl	5-butylpyridin-2-yl	89.7	3.5	3.4	1.0
18	5-Cl	5-phenylpyridin-2-yl	99.2	2.3	0.6	0.3
19	5-Cl	6-methylpyridin-2-yl	28.0	nc	nc	nc
20	5-Cl	quinoline-2-yl	25.9	nc	nc	nc
21	5-Cl	quinolin -3-yl	0.0	nc	nc	nc
22	5-Cl	quinolin -4-yl	88.9	2.3	16.1	6.9
23	5-Cl	isoquinolin-1-yl	53.7	nc	nc	nc
24	5-Cl	6-methoxy-quinolin-2-yl	22.3	nc	nc	nc
25	5-OCH_3_	pyridin-2-yl	50.9	nc	nc	nc
26	5-OCH_3_	5-methylpyridin-2-yl	93.5	3.3	11.1	3.4
27	5-OCH_3_	5-butylpyridin-2-yl	100.0	1.2	8.5	7.4
28	5-OCH_3_	5-phenylpyridin-2-yl	72.7	4.4	14.6	3.3
29	5-NO_2_	pyridin-2-yl	96.0	5.1	13.9	2.7
30	5-NH_2_	pyridin-2-yl	0.0	nc	nc	nc
31	5-NHCOC_9_H_18_(NHBoc)^c^	pyridin-2-yl	71.8	8.5	26.8	3.16
32	5-NHCOC_9_H_18_(NH_2_)^d^	pyridin-2-yl	72.3	6.8	16.41	2.43
33	5,6-diCH_3_	pyridin-2-yl	**100.0**	**0.85**	**13.2**	**15.4**
34	5,6-diCH_3_	5-butylpyridin-2-yl	74.2	3.4	3.2	1.0
35	5,6-diCH_3_	5-phenylpyridin-2-yl	64.7	7.3	9.7	1.3
36	5,6-diCl	pyridin-2-yl	81.9	2.4	11.8	5.0
Chloroquine			100.0	**0.028**	nc	nc

*nc* not calculated.

^a^Growth inhibition percentage (%) at 10 μM, ^b^cytotoxicity tested in Vero cells, ^c^2-(Boc-amino)decanamido, ^d^2-amino-decanamido.

Significant values are in bold.

**Figure 2:**
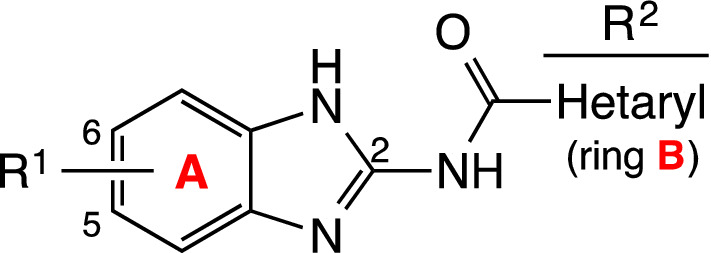
General structure of 2-amidobenzimidazole derivatives.

In Table 1 compounds are organized first according to the substituent on ring A, and then by ring B complexity (general structure: Fig. 2). Ring A displays electron donating groups, EDG, (5-CH_3_, 5-OCH_3_, 5-NH_2_, 5-NHCOR, or 5,6-diCH_3_) or electron withdrawing, EWG, (5-Cl, 5-NO_2_ or 5,6-diCl) in the BZ system, while ring B heterocycle can be a pyridine with the nitrogen in *ortho*, *meta* or *para* position, a quinoline or an isoquinoline. In addition, the presence of certain substituents (methyl, butyl, chloro, cyano, phenyl) on the pyridine ring has been explored, as well as its position.

Eleven derivatives (BZ **1** to **11**) displaying a methyl group on C-5 BZ were obtained. Compounds **1**, **2**, **3** showed in ring B a pyridine fragment with the nitrogen in 2′ (*ortho*), 3′(*meta*) or 4′(*para*) position. As observed in the results for antiplasmodial activity (Table 1) there is a clear preference for the *ortho* position since the picolinamide **1** reduced by 93.5% the growth of *Pf*, while the nicotinamide **2**, or the isonicotinamide **3** were inactive. BZ **1** showed an IC_50_ equal to 0.98 µM and an SI of 14.5. Then, the presence of certain substituents on the picolinamide fragment, their position (C-3′ or 5′) and electronical character (EWD, EDG) were explored, as well as the convenience of bulky fragments (quinoline, isoquinoline). EDG as methyl in C-3′ (IC_50_ = 2.5 µM for **4**) and specially in C-5´ (IC_50_ = 1.5 µM for **5**) are favorable, therefore new fragments in position C-5′ (Bu, Phe, CN) were introduced. The butyl derivative **6** with IC_50_ equal to 2.4 µM was slightly less potent than **5**, and the EWG 5-cyano **8** was inactive, electron rich fragments as the phenyl **7** were less potent than **5** (IC_50_ = 7.8 µµM). Of the bulkier groups (**9**, **10** and **11**) only the quinolin-2-yl **9** showed IC_50_ = 14.1 µM. These modifications, along with new others were introduced in combination with 5-Cl in ring A (BZ **12** to **24**). The effect of a methyl group in C-3´ (compound **13**) was similar to that of BZ **4** with IC_50_ = 2.8 µM and SI = 3.0, and an EWG as chloro (**14**) in that position was not suitable for the activity with only 11.9% GI of *Pf*. The combination 5-Cl/5′-Me (BZ **16**) was less efficient than the 5-Me/5′-Me (**5**), and the 5-Cl/6′-Me (**19**) was inactive. A methoxy group in position 4′ (BZ **15**) was introduced as EDG, which turned out to be the most potent compound of this group with IC_50_ = 1.5 µM, however, the picolinamide **12**, with a slightly lower activity, showed a higher SI of 8.7. In this case, of the bulkier fragments **20** to **24**, the quinolin-4-yl **22** showed good growth inhibitory effect with IC_50_ = 2.3 µM and SI = 6.9. Four derivatives with 5-OMe were synthesized (**25** to **28**); here the 5′-butylpyridin-2-yl **27** was the most potent with IC_50_ = 1.2 µM and SI = 7.4.

The 5-NO_2_/pyridin-2-yl **29** displayed moderate *Pf* inhibitory effect with IC_50_ = 5.1 µM. The activity was lost by reduction of the nitro group to an amino (BZ **30**), and recovered by modification to a long chain amide derivative, compounds **31** and **32**.

Disubstitution on ring A (5,6-diMe or (5,6-diCl) combined with pyridin-2-yl, 5-butylpyridin-2-yl, or 5-phenylpyridin-2-yl (BZ **33** to **36**) indicated that EDG associated with pyridin-2-yl (**33**) was an excellent combination, with IC_50_ = 0.85 µM and SI = 15.4, which is thirty times less active than the reference compound, chloroquine.

In general, and after the results showed in Table 1 we can state that the best association are EDG´s (one or two) in ring A combined with pyridine-2-yl in ring B.

### Cellular effect studies

**Figure 3:**
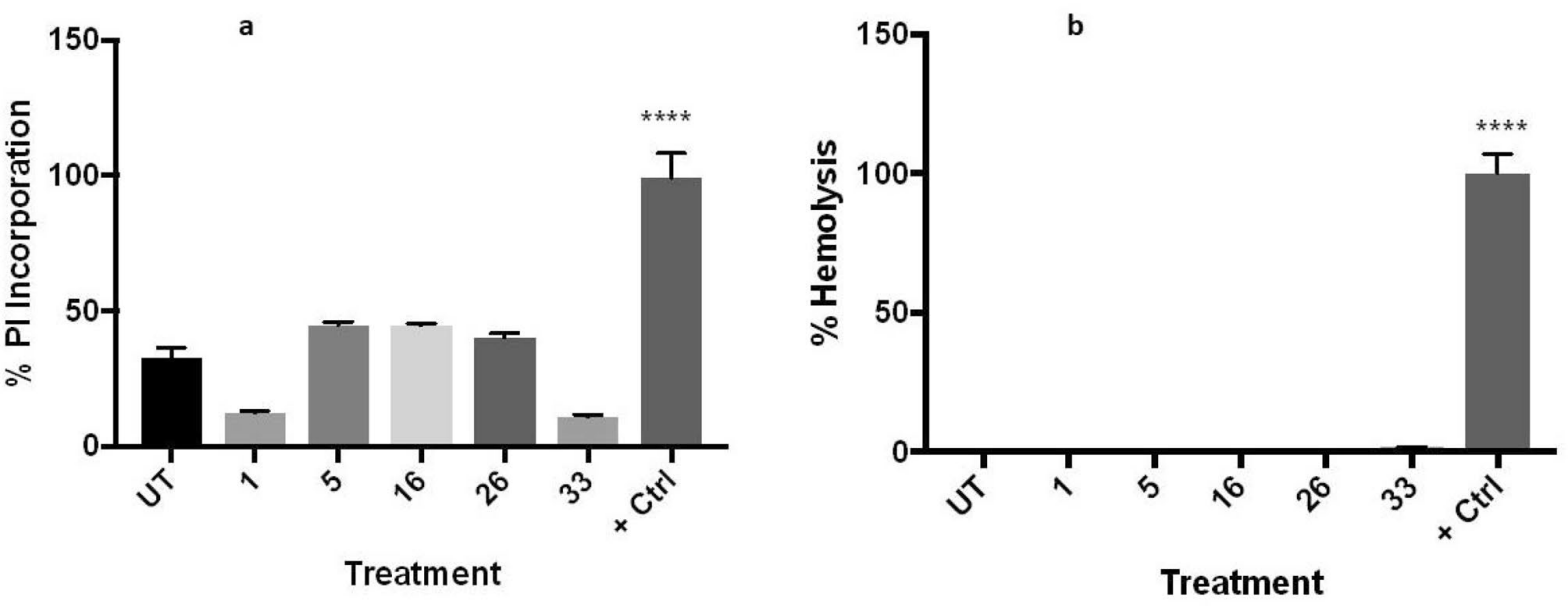
Membrane integrity tests. BZ **1**, **5**, **16**, **26** and **33** were tested in triplicates in two independent experiments. (**a**) Incorporation of propidium iodide (PI) was analyzed through fluorometry after 3 h incubation with the compounds. (**b**) Hemolysis was measured by light absorbance 24 h after exposure to the compounds. One-way ANOVA with Turkey´s comparison was used for analysis of significance. ****p < 0.001.

The integrity of the plasma membrane under the action of certain antiplasmodial compounds was assessed through the use of two methodologies. First, the absorption of propidium iodide, to which intact membranes are impermeable, was measured. Second, the presence of heme in the culture media, which indicates a leakage of the contents of the erythrocyte or its complete lysis (hemolysis), was analyzed. None of them showed any significant hemolysis action, however, some permeation in the membrane, although not significant, is caused by BZ **5**, **16** and **26**, while BZ **1** and **33** are the best in keeping the membrane well preserved (Fig. 3). This result is in agreement with the lower toxicity exhibited by the two latter with respect to the other three compounds.

**Figure 4:**
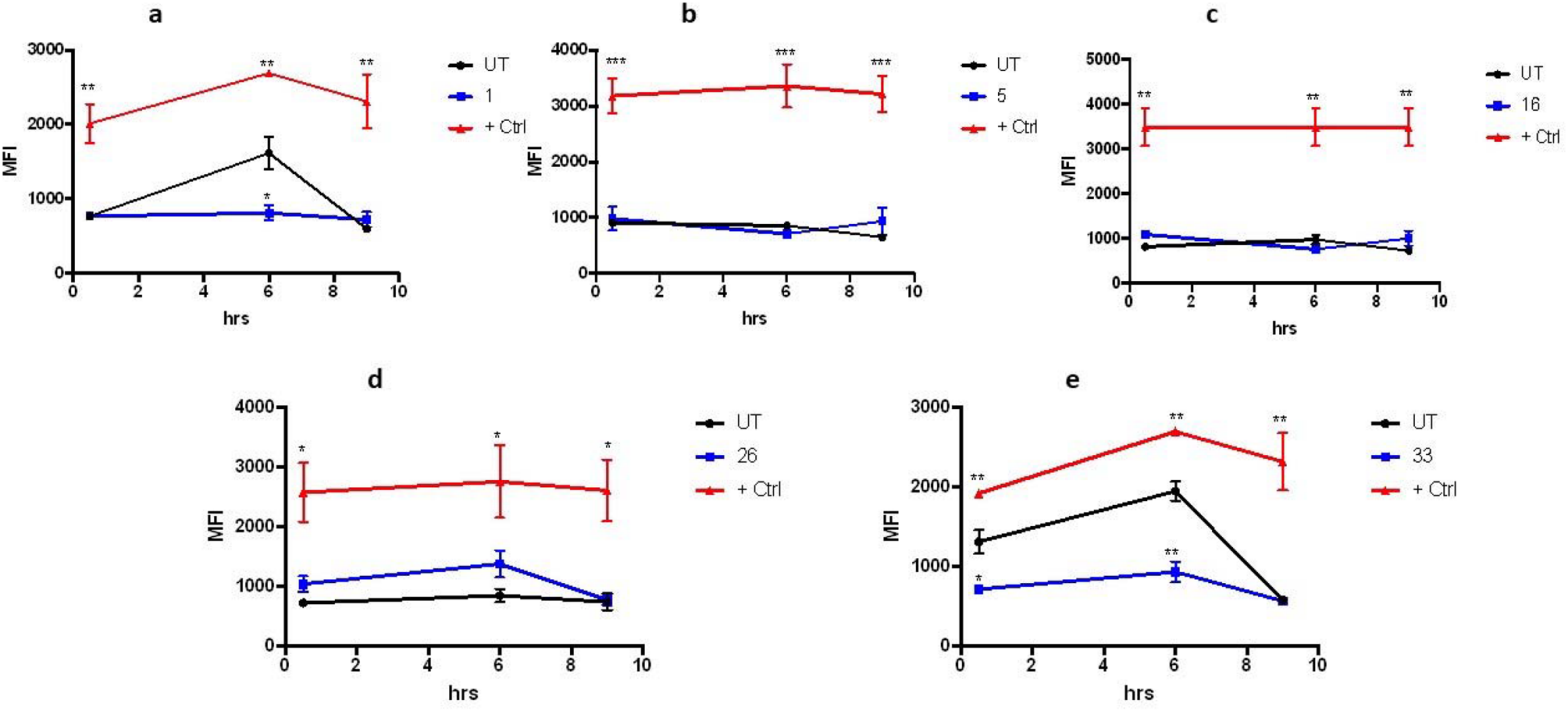
Effect of selected benzimidazole derivatives on ROS production. Five potent compounds were incubated with *P. falciparum*-infected cultures and the production of reactive oxygen species was measured through fluorometry at 0.5, 6 and 9 h after their addition at 1 µM. As positive control, a sample containing hydrogen peroxide at 200 μM was added. Samples were measured in triplicates. One-way ANOVA with Turkey´s comparison was used to measure significance with respect to the untreated control at each different time point. n = 2. (**a**) BZ **1**, (**b**) BZ **5**, (**c**) BZ **16**, (**d**) BZ **26** and (**e**) BZ **33.** *p < 0.05, **p < 0.01, ***p < 0.005.

Also, the intracellular production of oxygen reactive species (ROS) was monitored for the same antiplasmodial compounds. The level of ROS production was compared between the samples and that of the untreated parasites. Three compounds, BZ **5, 16** and **26,** showed no significant difference at any point. However, samples BZ **1** and **33** caused an early significant decrease in the natural level of ROS produced by *Pf* infection, which tapers off by t = 9 h (Fig. 4). This could explain why the latter, again, are the ones that are more effective in generating harm with the least toxicity given that they cause a transient shortage of oxidative reactions, which seem to last only enough to affect the parasite but not the epithelial Vero cells tested for cytotoxicity. This would point to the conformation of such molecules targeting a specific molecule or process of the parasite.

**Figure 5:**
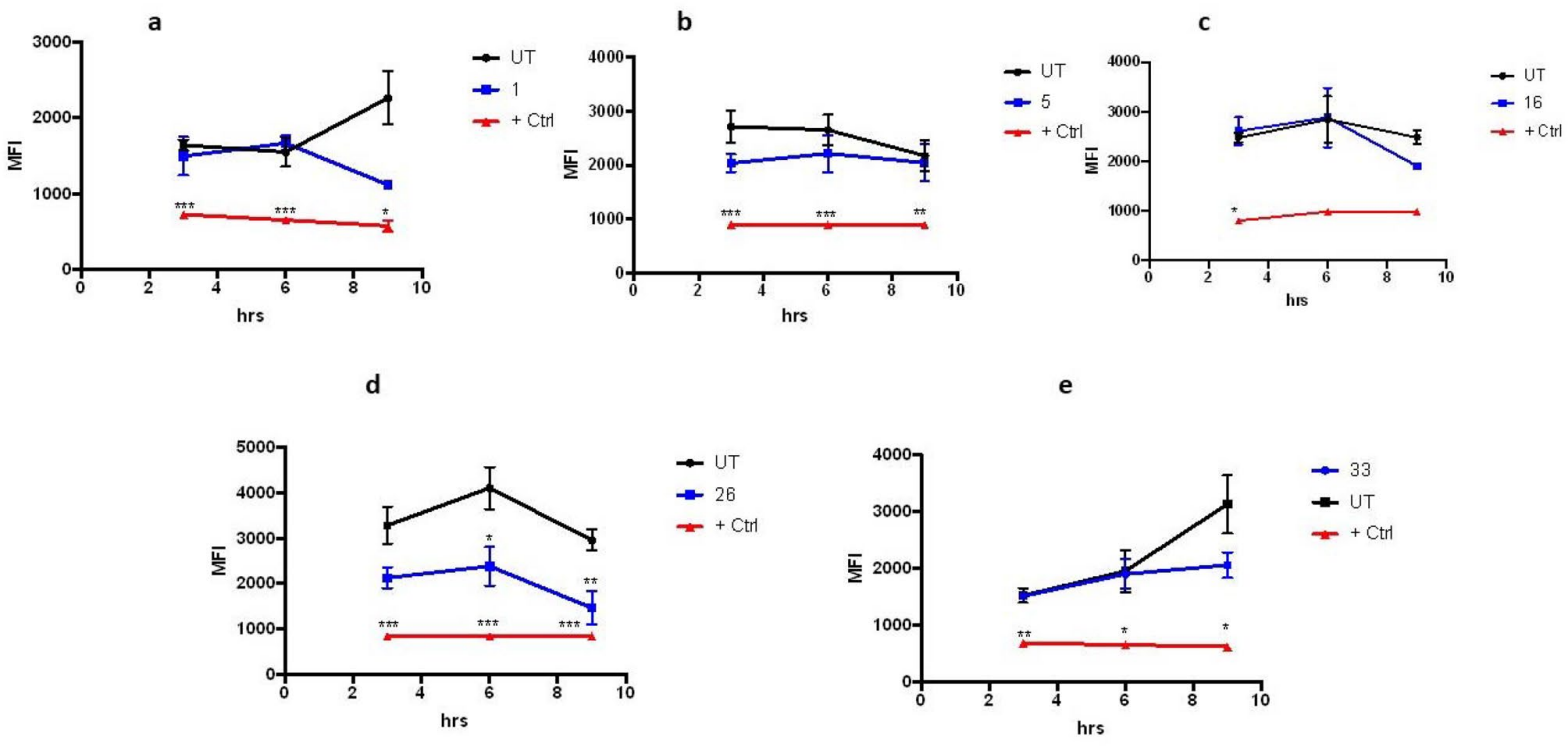
Changes to mitochondrial membrane potential caused by antiplasmodial compounds. Fluorometry was used to measure the effects on the mitochondria of *P. falciparum* parasites caused by treatment with compounds that show activity against their growth in culture. A membrane disruptor (CCCP) was added as a positive control and untreated cultures were used as the negative one. All compounds were tested at 1 µM in triplicates, n = 3. (**a**) BZ **1**, (**b**) BZ **5**, (**c**) BZ **16**, (**d**) BZ **26** and (**e**) BZ **33**. *p < 0.05, **p < 0.01, ***p < 0.005.

Changes in mitochondrial membrane potential, another common hallmark of programed cell death, were analyzed in the samples that had promising activities toward *P. falciparum* cultures. The results of adding 1 µM of each compound can be seen in Fig. 5. While four of the compounds tested do not show a significant departure from what happens to the mitochondrial membrane in the untreated controls, BZ **26** shows an effect from t = 6 h onward (Fig. 5), shifting toward the damaging effect of the positive control, which could perhaps be due to an additional interaction between the oxygen of the methoxy group attached to ring A with its specific target. Noteworthy, the two best antiplasmodial compounds, BZ **1** and BZ **33** do not show a difference with the untreated control up to t = 6 h, but there is a changing tendency at t = 9 h, that could suggest that only at that time those two compounds could start reaching the mitochondria. This could probably be explained by the presence of the unsubstituted picolinamide fragment, therefore more polar and less soluble in the membrane, making it harder or slower to permeate into the erythrocyte and then into the parasite, with respect to the other three.

**Figure 6:**
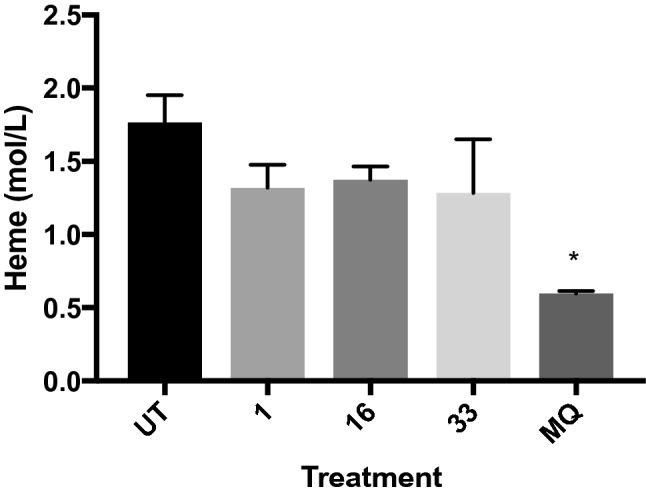
Influence in the biocrystallization of hemozoin in *P. falciparum*. Compounds **1, 16** and **33** were added to *Pf* cultures and the amount of hemozoin produced was analyzed after 36 h. Mefloquine (MQ) was used as a positive control. The experiment was performed in triplicates and analyzed with one-way ANOVA. *p < 0.05.

Finally, the interference of the BZ derivatives with hemozoin biocrystallization was measured in the two most promising samples (BZ **1** and **33**) together with one sample that was not selective towards *Pf* (BZ **16**) Fig. 6. The mechanism used by *Pf* to prevent intoxication with free heme once hemoglobin is degraded is to form hemozoin crystals that capture heme. Mefloquine and other common antimalarials interfere with this pathway, resulting in the death of the parasite. All the compounds tested here seem to interfere, but at a low level, with this process, but it clearly is not the main target of BZ **1** and BZ **33** against the parasite, given that a non-selective compound as BZ **16** gives the same, small level of disruption of the process as the other two. To explore a possible mechanism of action, three derivatives with different level of activities against *Pf* HB33 (BZ **1**, **12** and **16**) were also tested against *Pf* HB7G8, a strain that has developed chloroquine resistance (Table 2).

**Table 2:** Growth inhibition, IC_50,_ cytotoxicity and selectivity index of 2-amidobenzimidazole derivatives in 7G8 *P. falciparum* parasites.

Nº	R^1^	R^2^	7G8 *Pf* IC_50_ (µM)	Cytotoxicity^a^ CC_50_ (μM)	SI
1	5-CH_3_	pyridin-2-yl	0.95	14.8	15.6
12	5-Cl	pyridin-2-yl	3.60	15.4	4.2
16	5-Cl	5-methylpyridin-2-yl	2.60	7.2	2.7
CQ*			**0.23**	nc	nc

^a^cytotoxicity tested in Vero cells.

Table 2 shows that two of the compounds tested (**1** and **16**), unlike CQ, did not show an increase in their IC_50_ when confronted with a strain that has developed ways to detoxify from CQ, suggesting that some derivatives, as these two, might be targeting another pathway than CQ. On the other hand, the concentration of BZ **12** has to be doubled, with respect to *Pf* HB3, to achieve 50% killing in *Pf* 7G8, aligning with the notion that other BZ derivatives share a mechanism of action that participates at some point in the one used by chloroquine, possibly related with disturbing the ability of the parasite to synthetize hemozoin.

Significant values are in bold.

### ADME-toxicity profiles and physicochemical properties

In silico studies were performed through SwissADME (http://www.swissadme.ch/) and admetSAR (http://lmmd.ecust.edu.cn/admetsar2) online free servers to analyze the druggability and toxicity risks of most active compounds (Table 3). The most active BZs fulfill Lipinski’s Rule of Five and the Rule of Three of lead-likeness with MW values in the range of 252.27 and 286.72 g/mol, clogP values between 2.52 and 3.17, three H-bond acceptors for BZ **1**, **5**, **16** and **33** and four for BZ **26**, and H-donors in all of them equal to 2.

**Table 3:** ADMET properties of most active compounds against *Pf*.

Properties	BZ				
	1	5	16	26	33
MW	252.27	266.30	286.72	282.30	266.30
H-A^*a*^	3	3	3	4	3
H-D^*a*^	2	2	2	2	2
Log P	2.52	2.83	3.17	2.53	2.83
Rotatable bonds	2	2	2	3	2
TPSA	70.67	70.67	70.67	79.90	70.67
Drug-likeness	Yes	Yes	Yes	Yes	Yes
Lead-likeness	Yes	Yes	Yes	Yes	Yes
Log S	− 4.23	− 4.24	− 4.81	− 4.00	− 4.13
HIA	High	High	High	High	High
BBB	Yes	Yes	Yes	No	Yes
PPB	76.4	100.0	100	100	73.3
P-g substrate	Yes	No	No	Yes	Yes
Mutagenic	Yes	No	No	No	No
Tumorigenic	No	No	No	No	No

*MW* molecular weight, *Log P* lipophilicity, *PSA* topological polar surface area Å^2^, *Log S* aqueous solubility, *HIA* human intestinal absorption %, *BBB* blood–brain barrier, *PPB* plasma protein binding %.

^*a*^H-A Acceptor, H–D Donor.

Other properties as solubility, with values from − 4.81 to − 4.00 and topological polar surface area with 70.67 values for **1**, **5**, **16** and **33** and 79.90 for BZ **26** are favorable. Very good intestinal absorption, and blood brain barrier penetration with potential access to the central nervous system are shown in compounds **1**, **5**, **16** and **33.** This trait is especially necessary in their potential use to treat cerebral malaria, and is not predicted for BZ **26**, suggesting that the latter compound would work better in other manifestations of the infection. Plasma protein binding levels from 73.3 to 100% were predicted, which ensures a good distribution of the drug. None of the compounds showed mutagenic values except **1,** oddly enough given that compound **5**, which is structurally very similar, is not mutagenic. None of the compounds seem to be tumorigenic.

## Conclusions

A series of benzimidazole derivatives were synthetized and a structure–activity analysis performed, with the aim of improving their antiplasmodial activity. Some of the derivatives displayed IC_50s_ in the mid nanomolar range but were highly toxic. A number of mechanistic studies were conducted with a number of derivatives that had shown activity against the parasites, to try to understand the different response of the two best compound, BZ **1** and **33**, which showed high nanomolar activity with a good SI against *P. falciparum* parasites. Thus, for comparison reasons, these two lead compounds with nanomolar activity and selectivity index > 10 were analyzed together with other few molecules that were very active but, at the same time, toxic. None of the molecules showed a potent hemolytic activity; however, some of them caused harm to the membrane, which is an undesirable effect, that was diminished for BZ **1** and BZ **33**. In the production of reactive oxygen species and their effects on the potential of the mitochondrial membrane, BZ **1** and BZ **33** seem to differ from the other compounds tested, although in the interference with hemozoin crystallization they do not. These results, together, hint at a mechanism of action used by BZ **1** and BZ **33** that differ from the other compounds tested. This seems to be confirmed for **1,** when it made no difference to its IC_50_ to have it tested against a CQ-resistant strain. Compounds **1** and **33** displayed a methyl as electron donor group in ring A (one for **1** and two for **33**) and a non-substituted picolinamide fragment in ring B. These are characteristics that should be considered when trying to optimize their activity, which can be considered very good, given that the chloroquine activity differs from **1** and **33** only by some tenths of nano molarity and they seem to act through a different route than the signature antimalarial.

## Experimental

### Chemistry

#### General experimental procedures

All commercial chemicals (Aldrich, Alpha, Fischer, SDS) were used as purchased and solvents (Fischer, SDS, Scharlau) purified by the standard procedures prior to use^43^. Reactions were monitored by Thin Layer Chromatography (TLC) (Kieselgel 60 F254 precoated plates, E. Merck, Germany), the spots were detected by exposure to UV light at λ 254 nm, and colorization with 10% ninhydrin spray, and further heating of the plate. Melting points (mp) were determined with Mel-Temp apparatus in open capillaries and were uncorrected. Separations by flash column chromatography were performed on Merck 60 silica gel (0.063–0.2 mesh). NMR spectra were recorded on a Bruker Avance 400 MHz, Varian Mercury 400 MHz (400 MHz for ^1^H, 100 MHz for ^13^C) or Varian Mercury 200 MHz (200 MHz for ^1^H, 500 MHz for ^13^C). The spectra were measured either in CDCl_3_, methanol-d_6_ or DMSO-d_6_, using tetramethylsilane (TMS) as internal standard, chemical shifts (δ) are given in ppm and coupling constants (*J*) in Hertz. High resolution mass spectra (HRMS) were obtained by electron spray ionization-mass spectrometry (ESI–MS) technique (5 kV) on a QSTAR XL mass spectrometer.

### General procedures for the preparation of active compounds

#### Procedure for obtaining intermediate amino compounds, AIs 1–6

The obtaining process was the same as that described in Escala^40^; solutions of 1.00 g (6.53 mmol) 4-nitro-1,2-phenylendiamine and cyanogen bromide 1.04 g (9.80 mmol) in aqueous methanol (50% v/v) were prepared separately. The solutions were then mixed in an Erlenmeyer flask and maintained with continuous magnetic stirring at room temperature for 48 h, obtaining 1.10 g (6.20 mmol) 95%^41^.

*5-Nitro-1*H*-benzimidazol-2-amine* **AI 6**, orange solid; mp: 231 ºC; ^1^H NMR (400 MHz, CD_3_OD): δ 8.10 (d, *J* = 1.6 Hz, 1H), 8.07 (dd, *J* = 8.4 and 1.6 Hz, 1H), 7.43 (d, *J* = 8.4 Hz, 1H); ^13^C NMR (100 MHz, CD_3_OD): δ 153.90, 143.45, 136.39, 131.10, 118.91, 111.06, 106.89; HRMS (m/z): [M]^+^ calcd. for C_7_H_7_N_4_O_2_ [MS + H]^+^, 179.0491; found, 179.0410.

**AIs 1**–**5** were described in Escala^40^.

#### Coupling of intermediate amines with the corresponding acids. BZ 4, 5, 6, 13, 15, 16, 18, 22, 26, 27, 33 and 36

The protocol described in Escala et al.^40^ was applied. To a solution of the corresponding acid (1.3 mmol) in dry DMF (1 mL), DCI (1.3 mmol) was added, and the mixture maintained at room temperature for 1 h. Then, the corresponding 2-aminobenzimidazole intermediate (1.0 mmol) was added and the mixture maintained at room temperature with magnetic stirring for 16 h. The progress of the reaction was monitored by TLC using ethyl acetate as eluent. After completion of the reaction, the solvent was removed in vacuo in a rotary evaporator and the obtained solid was purified by silica gel chromatography using ethyl acetate as eluent. Reaction yields ranged between 30–69%.

*3-Methyl-*N*-(5-methyl-1*H*-benzimidazol-2-yl)picolinamide* **4**, a dark pink solid; mp: 173 °C; yield: 63%; ^1^H NMR (400 MHz, CDCl_3_): δ 11.00 (brs, 2H), 8.40 (d, *J* = 4.8 Hz, 1H), 7.61 (d, *J* = 7.6 Hz, 1H), 7.36 (dd, *J* = 7.6 and 4.8 Hz, 1H), 7.35 (d, *J* = 8.0 Hz, 1H), 7.25 (brs, 1H), 7.02 (dd, *J* = 8.0 and 1.2 Hz, 1H), 2.77 (s, 3H), 2.45 (s, 3H); ^13^C NMR (100 MHz, CDCl_3_): δ 165.0, 146.3, 146.0, 144.8, 141.2, 137.8, 136.7, 132.7, 131.8, 126.9, 123.4, 117.1, 110.8, 21.6, 20.5; HRMS (m/z): [M]^+^ calcd. for C_15_H_15_N_4_O [MS + H]^+^, 267.1240; found, 267.1236.

*5-Methyl-*N*-(5-methyl-1*H*-benzimidazol-2-yl)picolinamide* **5**, a white solid; mp: 256 °C; yield: 55%; ^1^H NMR (400 MHz, DMSO): δ 8.60 (d, *J* = 1.2 Hz, 1H), 8.11 (d, *J* = 7.6 Hz, 1H), 7.91 (dd, *J* = 7.6 and 1.2 Hz, 1H), 7.35 (d, *J* = 8.4 Hz, 1H), 7.28 (d, *J* = 1.2 Hz, 1H), 6.93 (dd, *J* = 8.4 and 1.2 Hz, 1H), 2.43 (s, 3H), 2.37 (s, 3H); ^13^C NMR (100 MHz, DMSO): δ 163.6, 149.6, 146.3, 145.9, 138.8, 138.3, 137.3, 132.9, 130.8, 123.1, 122.7, 114.3, 114.1, 21.7, 18.5; HRMS (m/z): [M]^+^ calcd. for C_15_H_15_N_4_O [MS + H]^+^, 267.1240; found, 267.1236.

*5-Butyl-*N*-(5-methyl-1*H*-benzimidazol-2-yl)picolinamide* **6**, a dark yellow solid, mp: 245 °C; yield: 52%; ^1^H NMR (400 MHz, CDCl_3_): δ 8.43 (d, *J* = 1.6 Hz, 1H), 8.18 (d, *J* = 8.0 Hz, 1H), 7.69 (dd, *J* = 8.0 and 1.6 Hz, 1H), 7.40 (d, *J* = 8.0 Hz, 1H), 7.30 (brs, 1H), 7.04 (brd, *J* = 8.0 Hz, 1H), 2.69 (t, *J* = 7.6 Hz, 2H), 2.46 (s, 3H), 1.63 (m, 2Η), 1.37 (m, 2H), 0.94 (t, *J* = 7.2 Hz, 3H); ^13^C NMR (100 MHz, CDCl_3_): δ 163.9, 148.9, 146.1, 145.7, 142.7, 137.1 (2C), 132.8, 131.9, 123.6, 122.5, 117.2, 116.8, 32.9, 32.7, 22.2, 21.6, 13.8; HRMS (m/z): [M]^+^ calcd. for C_18_H_21_N_4_O [MS + H]^+^, 309.1710; found, 309.1700.

N*-(5-Chloro-1*H*-benzimidazol-2-yl)-3-methylpicolinamide* **13**, an orange solid; mp: 180 °C; yield: 47%; ^1^H NMR (400 MHz, DMSO): δ 11.52 (brs, 2H), 8.38 (d, *J* = 4.0 Hz, 1H), 7.70 (d, *J* = 7.2 Hz, 1H), 7.41 (dd, *J* = 7.2 and 4.0 Hz, 1H), 7.34 (d, *J* = 2.0 Hz, 1H, 1H), 7.33 (d, *J* = 8.4 Hz, 1H), 6.98 (dd, *J* = 8.4 and 1.2 Hz, 1H), 2.49 (s, 3H); ^13^C NMR (100 MHz, DMSO): δ 165.0, 146.9, 146.9, 146.2, 140.7, 138.5, 134.6, 133.8, 126.7, 125.5, 121.3, 115.4, 114.2, 19.2; HRMS (m/z): [M]^+^ calcd. for C_14_H_12_N_4_OCl [MS + H]^+^, 287.0694; found, 287.0688.

N*-(5-Chloro-1*H*-benzimidazol-2-yl)-4-methoxypicolinamide* **15**, a light pink; mp: 245 °C; yield: 53%; ^1^H NMR (400 MHz, DMSO): δ 8.54 (d, *J* = 5.6 Hz, 1H), 7.67 (d, *J* = 2.4 Hz, 1H), 7.52 (d, *J* = 2.0 Hz, 1H), 7.49 (d, *J* = 8.4 Hz, 1H), 7.26 (dd, *J* = 5.6 and 2.4 Hz, 1H), 7.14 (dd, *J* = 8.4 and 2.0 Hz, 1H), 3.92 (s, 3H); ^13^C NMR (100 MHz, DMSO): δ 166.9, 163.1, 150.6, 150.0, 146.7, 139.5, 135.5, 125.8, 121.7, 114.0 (2C), 113.5, 109.0, 56.0; HRMS (m/z): [M]^+^ calcd. for C_14_H_12_N_4_O_2_Cl [MS + H]^+^, 303.0643; found, 303.0638.

N*-(5-Chloro-1*H*-benzimidazol-2-yl)-5-methylpicolinamide* **16**, an orange solid; mp: 201 °C; yield: 69%; ^1^H NMR (400 MHz, CDCl_3_): δ 8.46 (d, *J* = 1.2 Hz, 1H), 8.16 (d, *J* = 8.0 Hz, 1H), 7.73 (dd, *J* = 8.0 and 1.2 Hz, 1H), 7.51 (d, *J* = 1.6 Hz, 1H), 7.42 (d, *J* = 8.4 Hz, 1H), 7.20 (dd, *J* = 8.4 and 1.6 Hz, 1H), 2.46 (s, 3H); ^13^C NMR (100 MHz, CDCl_3_): δ 164.0, 149.3, 147.0, 145.0, 138.4, 138.0, 137.2, 134.8, 125.8, 122.8, 122.5, 114.8 (2C), 18.7; HRMS (m/z): [M]^+^ calcd. for C_14_H_12_N_4_OCl [MS + H]^+^, 287.0694; found, 287.0688.

N*-(5-Chloro-1*H*-benzimidazol-2-yl)-5-phenylpicolinamide* **18**, a white solid; mp: 214 °C; yield: 39%; ^1^H NMR (400 MHz, DMSO): δ 9.05 (d, *J* = 2.0 Hz, 1H), 8.37 (dd, *J* = 8.0 and 2.0 Hz, 1H), 8.27 (d, *J* = 8.0 Hz, 1H), 7.83 (dd, *J* = 8.6 and 1.6 Hz, 2H), 7.55 (m, 3H), 7.50 (d, *J* = 2.0 Hz, 1H), 7.48 (d, *J* = 8.4 Hz, 1H), 7.15 (dd, *J* = 8.4 and 2.0 Hz, 1H); ^13^C NMR (100 MHz, DMSO): δ 163.2, 148.6, 147.0, 146.8, 140.1, 139.9, 139.1, 135.9, 135.9, 129.3 (2C), 129.1, 127.3 (2C), 125.6, 123.0, 121.5, 116.2, 114.8; HRMS (m/z): [M]^+^ calcd. for C_19_H_14_N_4_OCl [MS + H]^+^, 349.0851; found, 349.0845.

N*-(5-Chloro-1*H*-benzimidazol-2-yl)quinoline-4-carboxamide* **22**, a yellow solid; mp: 262 °C; yield: 50%; ^1^H NMR (400 MHz, DMSO): δ 9.04 (d, *J* = 4.4 Hz, 1H), 8.32 (dd, *J* = 8.8 and 1.2 Hz, 1H), 8.13 (dd, *J* = 8.8 and 1.6 Hz, 1H), 7.85 (ddd, *J* = 8.8; 8.4 and 1.2 Hz, 1H), 7.83 (d, *J* = 4.4 Hz, 1H), 7.70 (ddd, *J* = 8.8; 8.4 and 1.6 Hz, 1H), 7.53 (d, *J* = 2.0 Hz, 1H), 7.50 (d, *J* = 8.4 Hz, 1H), 7.18 (dd, *J* = 8.4 and 2.0 Hz, 1H); ^13^C NMR (100 MHz, DMSO): δ 168.1, 150.6, 148.6, 148.3, 141.0, 137.3, 134.3, 130.5, 129.9, 128.2, 125.8 (2C), 124.4, 122.2, 120.5, 115.5, 114.2; HRMS (m/z): [M]^+^ calcd. for C_17_H_12_N_4_OCl [MS + H]^+^, 323.0694; found, 323.0888.

N*-(5-Methoxy-1*H*-benzimidazol-2-yl)-5-methylpicolinamide* **26**, a light yellow solid; mp: 213 °C; yield: 30%; ^1^H NMR (400 MHz, CDCl_3_): δ 8.47 (s, 1H), 8.14 (d, *J* = 8.0 Hz, 1H), 7.42 (d, *J* = 8.0 Hz, 1H), 7.25 (d, *J* = 8.4 Hz, 1H), 7.05 (brs, 1H), 6.89 (d, *J* = 8.0 Hz, 1H), 3.84 (s, 3H), 2.44 (3H, s); ^13^C NMR (100 MHz, CDCl_3_): δ 164.0, 155.7, 149.5, 145.7, 145.1, 138.3, 137.9, 136.5, 128.3, 122.5, 112.5, 111.9, 98.0, 55.9, 18.7; HRMS (m/z): [M]^+^ calcd. for C_15_H_15_N_4_O_2_ [MS + H]^+^, 283.1190; found, 283.1141.

*5-Butyl-N-(5-methoxy-1*H*-benzimidazol-2-yl)picolinamide* **27**, a dark yellow solid; mp: 129 °C; yield: 49%; ^1^H NMR (400 MHz, CDCl_3_): δ 10.84 (brs, 2H), 8.33 (d, *J* = 2.4 Hz, 1H), 8.16 (dd, *J* = 8.0 and 2.4 Hz, 1H), 7.65 (d, *J* = 8.0 Hz, 1H), 7.37 (d, *J* = 8.8 Hz, 1H), 7.00 (brs, 1H), 6.83 (dd, *J* = 8.8 and 1.2 Hz, 1H), 3.78 (s, 3H), 2.64 (t, *J* = 8.0 Hz, 2H), 1.59 (m, 2H), 1.31 (m, 2H), 0.90 (t, *J* = 7.2 Hz, 3H); ^13^C NMR (100 MHz, CDCl_3_): δ 163.8, 156.2, 148.7, 146.1, 145.6, 142.7, 137.2 (2C), 129.5, 122.6, 116.5, 111.2, 99.2, 55.8, 32.9, 32.8, 22.2, 13.8; HRMS (m/z): [M]^+^ calcd. for C_18_H_21_N_4_O_2_ [MS + H]^+^, 325.1659; found, 325.1652.

N*-(5,6-Dimethyl-1*H*-benzimidazol-2-yl)picolinamide* **33**, a yellow solid; mp: 221 °C; yield: 49%; ^1^H NMR (400 MHz, CDCl_3_): δ 11.0 (brs, 2H, D_2_O), 8.59 (dd, *J* = 4.8 and 1.2 Hz, 1H), 8.28 (d, *J* = 8.0 Hz, 1H), 7.90 (ddd, *J* = 8.0; 7.2 and 1.2 Hz, 1H), 7.50 (dd, *J* = 7.2 and 4.8 Hz, 1H), 7.28 (brs, 2H), 2.35 (s, 6H); ^13^C NMR (100 MHz, CDCl_3_): δ 163.5, 148.7, 148.0, 145.5, 137.7, 137.1 (2C), 131.0 (2C), 127.3, 122.7, 115.2 (2C), 20.3 (2C); HRMS (m/z): [M]^+^ calcd. for C_15_H_15_N_4_O [MS + H]^+^, 267.1240; found, 267.1235.

N*-(5,6-Dichloro-1*H*-benzimidazol-2-yl)picolinamide* **36**, a yellow solid; mp: 281 °C; yield: 46%; ^1^H NMR (400 MHz, DMSO): δ 12.60 (brs, 1H), 11.52 (brs, 1H), 8.77 (dd, *J* = 4.8 and 1.2 Hz, 1H), 8.21 (dd, *J* = 8.0 and 1.6 Hz, 1H), 8.11 (ddd, *J* = 8.0; 7.2 and 1.2 Hz, 1H), 7.74 (ddd, *J* = 7.2; 4.8 and 1.6 Hz, 1H), 7.72 (s, 2H); ^13^C NMR (100 MHz, DMSO): δ 163.5, 149.1, 148.1, 147.8, 138.5, 132.4 (2C), 128.0, 123.9 (2C), 123.0, 118.4 (2C); HRMS (m/z): [M]^+^ calcd. for C_13_H_9_N_4_OCl_2_ [MS + H]^+^, 307.0148; found, 307.0145.

BZs N*-(5-methyl-1*H*-benzimidazol-2-yl)picolinamide* **1**, N*-(5-chloro-1*H*-benzimidazol-2-yl)picolinamide* **12** were described in Escala^40^.

### Biology

#### Parasites and cultures

The chloroquine-sensitive strain HB3 and chloroquine-resistance 7G8 strain HB3 of *P. falciparum* were used throughout. Infected erythrocytes were kept in culture using the protocol established by Haynes et al.^44^, basically utilizing human O^+^ erythrocytes kept in an RPMI medium supplemented with glutamine and 10% human serum incubated in an atmosphere of 90% N_2_, 5% O_2_ and 5% CO_2_.

#### Hemolysis assay

Tests were performed in 96-well plates as described previously by Costa-Lotufo et al. The negative control contained only red blood cells in RPMI medium. All compounds were tested at 10 µM. A positive control, 20 µL of 0.1% Triton X-100 added to culture media, inducing 100% hemolysis, was used. Samples and controls were incubated at 37 °C. After 24 h, they were centrifuged collecting the supernatant which was read at 415 nm in a spectrophotometer.

#### Propidium iodide (PI) incorporation assay

Manufacturer’s instructions (BD PharmingenTM) were followed. Briefly, 200 µL of parasite culture at 2% parasitemia or uninfected erythrocytes, at 2% hematocrit, were incubated with each compound for 3 h at 37 °C. Untreated samples were cultured in the same plate. After incubation, 2 µL of Propidium Iodide was added and incubated for 15 min at RT while protected from light. Fluorescence was read in a fluorometer at excitation and emission wavelengths of 485/20 and 645/40 nm, respectively.

#### Mitochondrial membrane potential (Dy) measurements

The membrane potential was measured in a flow cytometer after staining *P. falciparum* cultures with the DiOC6(3) dye at 10 nM (Thermo Fisher, Waltham, MA, USA). Samples were incubated for 45 min at 37 °C in the dark. Following incubation, cells were washed and resuspended using 200 µL of phosphate-buffered saline (PBS) and analyzed immediately after. The mitochondrial membrane disrupter, CCCP (50 µM), incubated for 1 h, was used as a positive control. The fluorescence intensity of stained cells was calculated using FCS Express 4 (De Novo Software, Glendale, CA, USA). As negative control, uninfected red blood cells were used and their signal was subtracted from that of the infected red blood cells.

#### Intracellular reactive oxygen species (ROS) production in P. falciparum

Intracellular ROS formation was measured in a fluorimeter using the CM-H_2_DCFDA reactive dye from Molecular Probes® (Eugene OR, USA). Hydrogen peroxide was used as a positive control at a final concentration of 200 µM. The level of ROS present was inferred through the amount of oxidized DCF^45^.

#### In vitro β-hemozoin formation

The hemozoin polymerization assay was developed on the basis of the differential solubility of hemozoin and heme and was first used by Pandey et al. to quantify the amount of production of hemozoin by *Pf* parasites^46^. Using the methodology described by Tripathi et al.^47^, schizonts and late trophozoites at a parasitemia of 2–4% were used to obtain a parasite lysate. After overnight incubation with the samples, the absorbance of the final hemozoin pellet dissolved in NaOH was measured at 405 nm. Supernatants of soluble heme and hemozoin pellets were taken from washing steps and precipitated with concentrated (6 M) HCl. Pellets obtained were measured in a Fourier transformed infrared spectrometer (FT-IR) (Bruker, Germany) in attenuated total reflection (ATR) mode, at RT. The Beer-Lambert equation was used to calculate the final amount of hemozoin.

### Ethics statement

Human blood used in this study was collected from a pool of volunteers who signed informed consents. This protocol was approved by the bioethical committee of The Gorgas Memorial Institute of Health Sciences in Panama for this study through note 221/CBI/ICGES/21. All methods were performed in accordance with relevant guidelines and regulations.

## Supplementary Information

Supplementary Information.

## Data Availability

All the information required to reproduce the experiments (chemistry, activity assays and cellular activities) is included in the manuscript, and the rest in the Supplementary Material.
