# Supplementary material for: Antiplasmodial activity, structure–activity relationship and studies on the action of novel benzimidazole derivatives
Source: Sci Rep. 2023 Jan 6;13:285. doi: 10.1038/s41598-022-27351-z (PMC9822940; doi:10.1038/s41598-022-27351-z)
Supplement: Supplementary file 1 — Supplementary Information. [file 41598_2022_27351_MOESM1_ESM.pdf]

Supplementary Material

Antiplasmodial activity, structure-activity relationship and studies on the action of novel benzimidazole derivatives

Nerea Escala[1], Laura M. Pineda[2], Michelle G. Ng[2], Lorena M. Coronado[2], Carmenza Spadafora[2*] and Esther del Olmo[1*]

[1]Departamento de Ciencias Farmacéuticas: Química Farmacéutica, Facultad de Farmacia, Universidad de Salamanca, CIETUS, IBSAL, 37007-Salamanca, España.

[2]Center of Cellular and Molecular Biology of Diseases, Instituto de Investigaciones Científicas y Servicios de Alta Tecnología. City of Knowledge, Clayton, Apartado 0816-02852, Panama City, Panama.

*** Correspondence:**
Esther del Olmo
olmo@usal.es

Carmenza Spadafora
cspadafora@indicasat.org.pa

Table of contents

1. Description of synthetic final compounds and characterization of BZs **2**, **3**, **7**, **8**, **9**, **10**, **11**, **14**, **17**, **19**, **20**, **21**, **23**, **24**, **28**, **29**, **31**, **34**, **35**, **30** and **32**.
2. ^{1}H, ^{13}C and HRMS spectra of BZ **33**.

1. Description of synthetic final compounds and characterization.

The synthetic protocol was the same as the one described in Materials and Methods in the paper.

N-*(5-Methyl-1*H-*benzimidazol-2-yl)nicotinamide* **2**, a white solid; mp: 274 ºC; yield: 32%; ^{1}H NMR (400 MHz, DMSO): δ 9.38 (brs, 1H), 8.81 (d, *J* = 4.8 Hz, 1H), 8.53 (d, *J* = 8.0 Hz, 1H), 7.63 (dd, *J* = 8.0 and 4.8 Hz, 1H), 7.42 (d, *J* = 8.0 Hz, 1H), 7.35 (brs, 1H), 7.10 (brd, *J* = 8.0 Hz, 1H), 2.49 (s, 3H); ^{13}C NMR (100 MHz, DMSO): δ 169.65, 151.8, 151.0, 149.6, 135.8 (2C), 131.9, 131.7, 129.5, 123.3, 123.2, 112.3, 112.0, 21.2; HRMS (m/z): $[M]^+$ calcd. for $C_{14}H_{13}N_4O$ $[MS+H]^+$, 253.1084; found, 253.1079.

N-*(5-Methyl-1*H-*benzimidazol-2-yl)isonicotinamide* **3**, mp: 260 ºC; yield: 38%; ^{1}H NMR (400 MHz, DMSO): δ 12.45 (brs, 2H), 8.84 (d, *J* = 4.8 Hz, 2H), 8.10 (d, *J* = 4.8 Hz, 2H), 7.43 (d, *J* = 8.0 Hz, 1H), 7.35 (d, *J* = 1.6 Hz, 1H), 7.12 (dd, *J* = 8.0 and 1.6 Hz, 1H), 2.49 (s, 3H); ^{13}C NMR (100 MHz, DMSO): δ 170.1, 151.7, 150.1 (2C), 144.1, 131.0, 131.8, 123.8, 123.5, 122.2 (2C), 112.1, 111.9, 21.2; HRMS (m/z): $[M]^+$ calcd. for $C_{14}H_{13}N_4O$ $[MS+H]^+$, 253.1084; found, 253.1077.

N-*(5-Methyl-1*H-*benzimidazol-2-yl)-5-phenylpicolinamide* **7**, a light brown solid; mp: 170 ºC; yield: 30%; ^{1}H NMR (400 MHz, $CDCl_3$): δ 8.82 (brs, 1H), 8.31 (brd, *J* = 8.0 Hz, 1H), 8.07 (d, *J* = 8.0 Hz, 1H), 7.60 (d, *J* = 7.2 Hz, 2H), 7.50 (dd, *J* = 7.6 and 7.2 Hz, 2H), 7.47 (m, 1H), 7.43 (d, *J* = 8.0 Hz, 1H), 7.33 (brs, 1H), 7.08 (brd, *J* = 8.0 Hz, 1H), 2.47 (s, 3H); ^{13}C NMR (100 MHz, $CDCl_3$): δ 163.7, 147.3, 146.2, 145.8, 140.4, 137.8, 136.3, 135.6, 132.7, 132.6, 129.3 (2C), 129.1, 127.3 (2C), 124.1, 122.9, 114.1, 113.9, 21.6; HRMS (m/z): $[M]^+$ calcd. for $C_{20}H_{17}N_4O$ $[MS+H]^+$, 329.1397; found, 329.1484.

*5-Cyano-*N-*(5-methyl-1*H-*benzimidazol-2-yl)picolinamide* **8**, a dark yellow solid; mp: 214 ºC; yield: 42%; ^{1}H NMR (200 MHz, CD_3OD): δ 9.07 (dd, *J* = 2.0 and 0.8 Hz, 1H), 8.43 (dd, *J* = 8.4 and 2.0 Hz, 1H), 8.43 (d, *J* = 8.0 Hz, 1H), 7.37 (d, *J* = 8.0 Hz, 1H), 7.32 (brs, 1H), 7.06 (brd, *J* = 8.0 Hz, 1H), 2.45 (s, 3H); ^{13}C NMR (100 MHz, CD_3OD): δ 162.2, 153.4, 152.8, 147.5, 142.9, 138.0, 133.3, 132.7, 126.0, 125.0, 124.0, 123.0, 117.1, 113.7, 21.7; HRMS (m/z): $[M]^+$ calcd. for $C_{15}H_{12}N_5O$ $[MS+H]^+$, 278.1036; found, 278.1031.

N-*(5-Methyl-1*H-*benzimidazol-2-yl)quinoline-2-carboxamide* **9**, a yellow solid; mp: 195 ºC; yield: 55%; ^{1}H NMR (400 MHz, $CDCl_3$): δ 10.99 (brs, 1H), 8.41 (d, *J* = 8.4 Hz, 1H), 8.35 (t, *J* = 8.4 Hz, 1H), 8.16 (d, *J* = 8.4 and 2.0 Hz, 1H), 7.92 (dd, *J* = 8.4 and 1.4 Hz, 1H), 7.85 (dt, *J* = 8.4 and 2.0 Hz, 1H), 7.70 (dt, *J* = 8.4 and 1.4 Hz, 1H), 7.44 (d, *J* = 8.8 Hz, 1H), 7.35 (brs,

1H), 7.08 (brd, J = 8.8 Hz, 1H), 2.49 (s, 3H); ^{13}C NMR (100 MHz, $CDCl_3$): δ 163.7, 147.6, 147.4, 146.5, 140.8, 138.7, 138.1, 132.2, 130.3, 130.0, 129.8, 128.9, 127.7, 121.0, 118.6, 118.0, 110.5, 21.7; HRMS (m/z): $[M]^+$ calcd. for $C_{18}H_{15}N_4O$ $[MS+H]^+$, 303.1240; found, 303.1234.

N-*(5-Methyl-1*H-*benzimidazol-2-yl)quinoline-4-carboxamide* **10**, a yellow solid; mp: 205 ºC; yield: 59%; ^{1}H NMR (400 MHz, DMSO): δ 8.66 (d, J = 8.4 Hz, 1H), 8.29 (dd, J = 7.2 and 1.0 Hz, 1H), 8.25 (d, J = 8.4 Hz, 1H), 8.14 (dd, J = 7.2 and 1.2 Hz, 1H), 7.91 (dt, J = 7.2 and 1.2 Hz, 1H), 7.77 (dt, J = 7.2 and 1.0 Hz, 1H), 7.37 (d, J = 8.0 Hz, 1H), 7.30 (d, J = 1.2 Hz, 1H), 6.96 (dd, J = 8.0 and 1.2 Hz, 1H), 2.39 (s, 3H); ^{13}C NMR (100 MHz, DMSO): δ 164.0, 149.1, 146.4, 146.1, 138.8, 136.9, 135.7, 131.3, 131.0, 130.0, 129.6, 129.2, 128.5, 123.2, 119.1, 114.9 (2C), 21.7; HRMS (m/z): $[M]^+$ calcd. for $C_{18}H_{15}N_4O$ $[MS+H]^+$, 303.1240; found, 303.1232.

N-*(5-Methyl-1*H-*benzimidazol-2-yl)isoquinoline-1-carboxamide* **11**, a yellow solid; mp: 190 ºC; yield: 57%; ^{1}H NMR (400 MHz, DMSO): δ 12.32 (brs, 2H), 8.94 (d, J = 8.4 Hz, 1H), 8.72 (d, J = 5.2 Hz, 1H), 8.22 (d, J = 8.0 Hz, 1H), 8.20 (d, J = 5.6 Hz, 1H), 7.98 (dt, J = 8.4 and 8.0 Hz, 1H), 7.90 (t, J = 8.0 Hz, 1H), 7.47 (d, J = 8.4 Hz, 1H), 7.38 (s, 1H), 7.06 (d, J = 8.4 Hz, 1H), 2.49 (s, 3H); ^{13}C NMR (100 MHz, $CDCl_3$): δ 165.4, 146.6, 146.3, 142.7, 140.4, 137.3, 134.5, 131.8, 130.6, 129.1, 127.1, 127.0, 126.8, 125.3, 123.5, 114.1, 113.9, 21.6; HRMS (m/z): $[M]^+$ calcd. for $C_{18}H_{15}N_4O$ $[MS+H]^+$, 303.1240; found, 303.1234.

*3-Chloro-N-(5-chloro-1*H-*benzimidazol-2-yl)picolinamide* **14**, a white solid; mp: 241 ºC; yield: 47%; ^{1}H NMR (400 MHz, DMSO): δ 8.63 (dd, J = 4.8 and 1.2 Hz, 1H), 8.12 (dd, J = 8.4 and 1.2 Hz, 1H), 7.63 (dd, J = 8.4 and 4.8 Hz, 1H), 7.51 (d, J = 2.0 Hz, 1H), 7.48 (d, J = 8.4 Hz, 1H), 7.15 (dd, J =8.4 and 2.0 Hz, 1H); ^{13}C NMR (100 MHz, DMSO): δ 164.5, 149.4, 147.7, 147.4, 138.7, 138.5, 136.5, 128.6, 126.9, 125.7, 121.5, 114.5 (2C); HRMS (m/z): $[M]^+$ calcd. for $C_{13}H_9N_4OCl_2$ $[MS+H]^+$, 307.0148; found, 307.0144.

*5-Butyl-N-(5-chloro-1*H-*benzimidazol-2-yl)picolinamide* **17**, a light pink solid; mp: 132 ºC; yield: 62%; ^{1}H NMR (400 MHz, DMSO): δ 11.34 (brs, 1H), 8.58 (d, J = 2.0 Hz, 1H), 8.13 (d, J = 7.8 Hz, 1H), 7.92 (dd, J =7.8 and 2.0 Hz, 1H), 7.51 (d, J = 2.0 Hz, 1H), 7.49 (d, J = 8.6 Hz, 1H), 7.13 (dd, J = 8.6 and 2.0 Hz, 1H), 2.69 (t, J = 7.4 Hz, 2H), 1.59 (m, 2H), 1.30 (m, 2H), 0.88 (t, J = 7.0 Hz, 3H); ^{13}C NMR (100 MHz, DMSO): δ 163.2, 148.9, 146.7, 145.9, 142.5, 139.5, 137.7, 132.6, 125.5, 122.5, 121.4, 116.5, 112.5, 32.4, 31.7, 21.6, 13.6; HRMS (m/z): $[M]^+$ calcd. for $C_{17}H_{18}N_4OCl$ $[MS+H]^+$, 329.1164; found, 329.1157.

N-*(5-Chloro-1*H-*benzimidazol-2-yl)-6-methylpicolinamide* **19**, a light brown solid; mp: 182 ºC; yield: 58%; ^{1}H NMR (400 MHz, DMSO): δ 11.14 (brs, 2H), 7.89 (d, J = 5.4 Hz, 1H), 7.86 (dd, J = 7.8 and 5.4 Hz, 1H), 7.48 (d, J = 7.8 Hz, 1H), 7.39 (d, J = 2.0 Hz, 1H), 7.36 (d, J = 8.6 Hz, 1H), 7.02 (dd, J = 8.6 and 2.0 Hz, 1H), 2.36 (s, 3H); ^{13}C NMR (100 MHz, DMSO): δ 163.1,

157.8, 147.2, 146.6, 140.1, 138.5, 133.8, 127.5, 125.6, 121.5, 119.8, 114.6, 112.4, 23.7; HRMS (m/z): $[M]^+$ calcd. for $C_{14}H_{12}N_4OCl$ $[MS+H]^+$, 287.0694; found, 287.0688.

N-*(5-Chloro-1*H-*benzimidazol-2-yl)quinoline-2-carboxamide* **20**, a yellow solid; mp: 211 ºC; yield: 56%; ^{1}H NMR (400 MHz, DMSO): δ 11.66 (brs, 1H), 8.69 (d, *J* = 7.6 Hz, 1H), 8.29 (d, *J* = 8.4 Hz, 1H), 8.29 (d, *J* = 8.4 Hz, 1H), 8.27 (d, *J* = 7.6 Hz, 1H), 7.93 (dd, *J* = 8.4 and 7.6 Hz, 1H), 7.78 (t, *J* = 8.4 Hz, 1H), 7.55 (d, *J* = 2.0 Hz, 1H), 7.51 (d, *J* = 8.4, 1H), 7.16 (dd, *J* = 8.4 and 2.0 Hz, 1H); ^{13}C NMR (100 MHz, DMSO): δ 163.5, 148.2, 146.7, 146.0, 141.2, 138.5, 131.5, 130.9, 129.6, 129.5, 128.9, 128.1, 125.6, 121.5, 118.6, 112.0 (2C); HRMS (m/z): $[M]^+$ calcd. for $C_{17}H_{12}N_4OCl$ $[MS+H]^+$, 323.0694; found, 323.0687.

N-*(5-Chloro-1*H-*benzimidazol-2-yl)quinoline-3-carboxamide* **21**, a light brown solid; mp: 312 ºC; yield: 47%; ^{1}H NMR (400 MHz, DMSO): δ 12.50 (brs, 1H), 9.49 (d, *J*=2.4 Hz, 1H), 9.08 (d, *J* = 2.4 Hz, 1H), 8.15 (dd, *J* = 7.6 and 1.2 Hz, 1H), 8.10 (dd, *J* = 8.0 and 1.2 Hz, 1H), 7.90 (ddd, *J* = 8.0; 7.2 and 1.2 Hz, 1H), 7.70 (dt, *J* = 8.0 and 1.2 Hz, 1H), 7.50 (s, *J* = 2.0 Hz, 1H), 7.47 (d, *J* = 8.0 Hz, 1H), 7.18 (dd, *J* = 8.0 and 2.0 Hz, 1H); ^{13}C NMR (100 MHz, DMSO): δ 167.8, 150.0, 149.2, 149.1, 137.5, 135.8, 133.2, 132.1, 129.9, 129.1, 127.9, 127.7, 126.9, 126.4, 122.3, 114.9, 113.6; HRMS (m/z): $[M]^+$ calcd. for $C_{17}H_{12}N_4OCl$ $[MS+H]^+$, 323.0694; found, 323.0690.

N-*(5-Chloro-1*H-*benzimidazol-2-yl)isoquinoline-1-carboxamide* **23**, a light pink; mp: 225 ºC; yield: 64%; ^{1}H NMR (400 MHz, DMSO): δ 8.84 (d, *J* = 8.4 Hz, 1H), 8.64 (d, *J* = 5.6 Hz, 1H), 8.13 (d, *J* = 5.6 Hz, 1H), 8.11 (d, *J* = 7.2 Hz, 1H), 7.87 (dd, *J* = 8.4 and 7.2 Hz, 1H), 7.81 (t, *J* = 7.2 Hz, 1H), 7.53 (d, *J* = 2.0 Hz, 1H), 7.50 (d, *J* = 8.4 Hz, 1H), 7.16 (dd, *J* = 8.4 and 2.0 Hz, 1H); ^{13}C NMR (100 MHz, DMSO): δ 165.5, 149.4, 147.0, 141.1, 137.5, 136.6, 133.2, 131.0, 129.0, 127.4, 125.7, 125.6, 125.5, 124.3, 121.4, 115.7, 114.8; HRMS (m/z): $[M]^+$ calcd. for $C_{17}H_{12}N_4OCl$ $[MS+H]^+$, 323.0694; found, 323.0687.

N-*(5-Chloro-1*H-*benzimidazol-2-yl)-6-methoxyquinoline-2-carboxamide* **24**, a dark red solid; mp: 335 ºC; yield: 44%; ^{1}H NMR (400 MHz, DMSO): δ 9.33 (d, *J* = 2.0 Hz, 1H), 8.94 (d, *J* = 2.0 Hz, 1H), 8.00 (d, *J* = 8.0 Hz, 1H), 7.51 (d, *J* = 2.0 Hz, 1H), 7.48 (d, *J* = 8.0 Hz, 1H), 7.48 (d, *J* = 2.0 Hz, 1H), 7.47 (dd, *J* = 8.0 and 2.0 Hz, 1H), 7.18 (dd, *J* = 8.0 and 2.0 Hz, 1H), 3.93 (s, 3H); ^{13}C NMR (100 MHz, DMSO): δ 163.2, 157.8 (2C), 147.1, 144.9, 140.1, 136.1, 135.7, 130.2 (2C), 127.8, 125.9, 124.1, 121.8, 116.1, 115.8, 106.9, 55.7; HRMS (m/z): $[M]^+$ calcd. for $C_{18}H_{14}N_4O_2Cl$ $[MS+H]^+$, 353.0800; found, 353.0790.

N-*(5-Methoxy-1*H-*benzimidazol-2-yl)-5-phenylpicolinamide* **28**, a light yellow solid; mp: 180 ºC; yield: 27%; ^{1}H NMR (400 MHz, $CDCl_3$): δ 8.87 (d, *J* = 2.4 Hz, 1H), 8.32 (d, *J* = 8.4 Hz, 1H), 8.09 (dd, *J* = 8.4 and 2.4 Hz, 1H), 7.63 (dd, *J* = 7.2 and 1.6 Hz, 2H), 7.50 (t, *J* = 7.2 Hz,

2H), 7.49 (dd, *J* = 7.2 and 1.6 Hz, 1H), 7.43 (d, *J* = 8.4 Hz, 1H), 7.07 (d, *J* = 2.0 Hz, 1H), 6.90 (dd, *J* = 8.4 and 2.0 Hz, 1H), 3.85 (s, 3H); ^{13}C NMR (100 MHz, $CDCl_3$): δ 163.7, 156.7, 147.4, 146.1, 145.7, 140.5, 136.3, 135.7, 135.5, 129.4 (2C), 129.3, 129.2, 127.3 (2C), 123.0, 111.9, 108.0, 98.1, 55.9; HRMS (m/z): $[M]^+$ calcd. for $C_{20}H_{17}N_4O_2$ $[MS+H]^+$, 345.1273; found, 345.1312.

N-*(5-Nitro-1*H-*benzimidazol-2-yl)picolinamide* **29**, a white solid; mp: 282 ºC; yield: 42%; ^{1}H NMR (400 MHz, DMSO): δ 12.91 (brs, 1H), 11.67 (brs, 1H), 8.73 (dd, *J* = 4.8 and 2.0 Hz, 1H), 8.35 (brs, 1H), 8.20 (dd, *J* = 7.6 and 0.8 Hz, 1H), 8.09 (ddd, *J* = 7.6; 7.2 and 2.0, 1H), 8.04 (d, *J* = 8.4 Hz, 1H), 7.71 (ddd, *J* = 7.2; 4.8 and 0.8 Hz, 1H), 7.62 (brd, *J* = 8.4 Hz, 1H); ^{13}C NMR (100 MHz, DMSO): δ 164.1, 159.5, 150.3, 149.4, 148.4, 145.5, 142.2, 138.8, 128.4, 123.4, 117.9, 112.0, 108.6; HRMS (m/z): $[M]^+$ calcd. for $C_{13}H_{10}N_5O_3$ $[MS+H]^+$, 284.0778; found, 284.0773.

*Tert-butyl (1-oxo-1-((2-(picolinamido)-1*H-*benzimidazol-5-yl)amino)decan-2-yl) carbamate* **31**, yellow oil; yield: 32%; ^{1}H NMR (400 MHz, $CDCl_3$): δ 8.65 (d, *J* = 4.4 Hz, 1H), 8.46 (brs, 1H), 8.26 (d, *J* = 8.0 Hz, 1H), 7.92 (dd, *J* = 8.0 and 7.2 Hz, 1H), 7.90 (d, *J* = 8.8 Hz, 1H), 7.52 (dd, *J* = 7.2 and 4.4 Hz, 1H), 7.44 (d, *J* = 8.8 Hz, 1H), 7.20 (brs, 1H), 5.27 (d, *J* = 7.6 Hz, 1H), 5.22 (d, *J* = 8.0 Hz, 1H), 4.23 (m, 1H), 1.96 (m, 2H), 1.70 (m, 2H), 1.44 (s, 9H), 1.27 (m, 10H), 0.87 (t, *J* = 6.4 Hz, 3H); ^{13}C NMR (100 MHz, $CDCl_3$): δ 170.6, 164.0, 155.9, 148.8, 147.9, 146.4, 137.6, 133.5, 133.1, 130.2, 127.4, 122.9, 118.2, 115.9, 103.1, 79.9, 53.9, 31.8, 29.9, 29.2, 28.4, 28.3 (3C), 25.8, 25.3, 22.6, 14.1; HRMS (m/z): $[M]^+$ calcd. for $C_{28}H_{39}N_6O_4$ $[MS+H]^+$, 523.3027; found, 523.3021.

*5-Butyl-*N-*(5,6-dimethyl-1*H-*benzimidazol-2-yl)picolinamide* **34**, a light yellow solid; mp: 167 ºC; yield: 41%; ^{1}H NMR (400 MHz, $CDCl_3$): δ 10.40 (brs, 1H), 8.36 (brs, 1H), 8.16 (d, *J* = 7.6 Hz, 1H), 7.65 (brd, *J* = 7.6 Hz, 1H), 7.26 (s, 2H), 2.66 (m, 2H), 2.33 (s, 6H), 1.60 (m, 2H), 1.35 (m, 2H), 0.93 (t, *J* = 7.6 Hz, 3H); ^{13}C NMR (100 MHz, $CDCl_3$): δ 163.8, 148.9, 148.9, 145.7, 142.7, 137.2, 136.1 (2C), 131.0 (2C), 122.5, 117.3 (2C), 32.9, 32.8, 22.2, 20.3 (2C), 13.8; HRMS (m/z): $[M]^+$ calcd. for $C_{19}H_{23}N_4O$ $[MS+H]^+$, 323.1872; found, 323.1880.

N-*(5,6-Dimethyl-1*H-*benzimidazol-2-yl)-5-phenylpicolinamide* **35**, a brown solid; mp: 241 ºC; yield: 35%; ^{1}H NMR (400 MHz, $CDCl_3$): δ 8.77 (brs, 1H), 8.25 (d, *J* = 7.2 Hz, 1H), 8.02 (brd, *J* = 7.2 Hz, 1H), 7.55 (d, *J* = 6.4 Hz, 2H), 7.43 (d, *J* = 6.4 Hz, 2H), 7.41 (m, 1H), 7.24 (s, 2H), 2.29 (s, 6H); ^{13}C NMR (100 MHz, $CDCl_3$): δ 163.6, 147.3, 147.3, 146.4, 140.4, 136.4, 135.7 (2C), 135.7, 131.4 (2C), 129.3 (3C), 127.3 (2C), 122.8, 114.9 (2C), 20.3 (2C); HRMS (m/z): $[M]^+$ calcd. for $C_{21}H_{19}N_4O$ $[MS+H]^+$, 343.1559; found, 343.1552.

N-*(5-methoxy-1*H-*benzimidazol-2-yl)picolinamide* **25** was described in Escala et al.[40].

Procedure for the reduction of the nitrobenzimidazole 29.

The obtaining process was described in Escala[40], 100 mg (0.371 mmol) of BZ **29** were dissolved in MeOH (3 mL) placed in a two-necked round bottom flask, then, Pd-C catalyst (3 mg) was added, and H_2 atmosphere generated. The mixture was maintained at room temperature for 2 h. After the reaction was complete, the Pd-C was removed by filtration through Celite, and the solvent was removed in vacuo to give a yellow solid that was purified by column chromatography to provide 75 mg (85%) of compound **30**.

N-*(5-Amino-1*H-*benzimidazol-2-yl)picolinamide* **30**, mp: 235 ºC; ^{1}H NMR (400 MHz, CD_3OD): δ 8.67 (d, *J* = 4.4 Hz, 1H), 8.25 (d, *J* = 7.0 Hz, 1H), 7.98 (t, *J* = 7.6 Hz, 1H), 7.58 (dd, *J* = 7.6 and 4.4 Hz, 1H), 7.24 (d, *J* = 8.4 Hz, 1H), 6.88 (d, *J* = 1.2 Hz, 1H), 6.69 (dd, *J* = 8.4 and 1.2 Hz, 1H); ^{13}C NMR (100 MHz, CD_3OD): δ 165.3, 154.9, 149.2, 147.2, 140.4, 137.9, 137.6, 127.2, 125.0, 122.6, 111.4, 109.6, 100.0; HRMS (m/z): $[M]^+$ calcd. for $C_{13}H_{12}N_5O$ $[MS+H]^+$, 254.1036; found, 254.1030.

Synthesis of compound 32 by the deprotection of the Boc group in compound 31.

The process was described in Escala[40], 40 mg (0.074 mmol) of BZ **31** were dissolved in CH_2Cl_2 (3 mL) and kept at 0 °C under stirring. Then, trifluoroacetic acid (0.6 mL) was added, and the reaction monitored every 15 min by running TLC [eluent: *n*-hexane / ethyl acetate (1:9)]. After the reaction has finished, it was extracted with CH_2Cl_2 and washed with water. The solvent was removed under vacuum on a rotary evaporator to provide BZ **32** (10 mg) in 76% yield.

N-*(5-(2-Aminodecanamido)-1*H-*benzimidazol-2-yl)picolinamide* **32**, a yellow oil; yield: 76 %; ^{1}H NMR (400 MHz, $CDCl_3$): δ 9.57 (brs, 1H), 8.62 (dd, *J* = 4.4 and 1.6 Hz, 1H), 8.28 (d, *J* = 7.6 Hz, 1H), 8.10 (m, 1H), 7.93 (dt, *J* = 7.6 and 1.6 Hz, 1H), 7.52 (dd, *J* = 7.6 and 4.4 Hz, 1H), 7.48 (brs, 1H), 7.10 (brs, 1H), 3.52 (m, 1H), 2.00 (m, 2H), 1.64 (m, 2H), 1.46 (m, 2H), 1.26 (m, 8H), 0.79 (t, *J* = 6.8 Hz, 3H); ^{13}C NMR (100 MHz, $CDCl_3$): δ 173.1, 163.5, 148.7, 147.8, 146.4, 137.9, 137.7, 133.0, 132.7, 127.4, 122.8, 118.1, 115.1, 102.3, 55.7, 35.0, 29.7, 29.4, 29.3, 29.2, 25.7, 22.6, 14.1; HRMS (m/z): $[M]^+$ calcd. for $C_{23}H_{31}N_6O_2$ $[MS+H]^+$, 423.2503; found, 423.2495.

2. ¹H, ¹³C and HRMS spectra of BZ 33

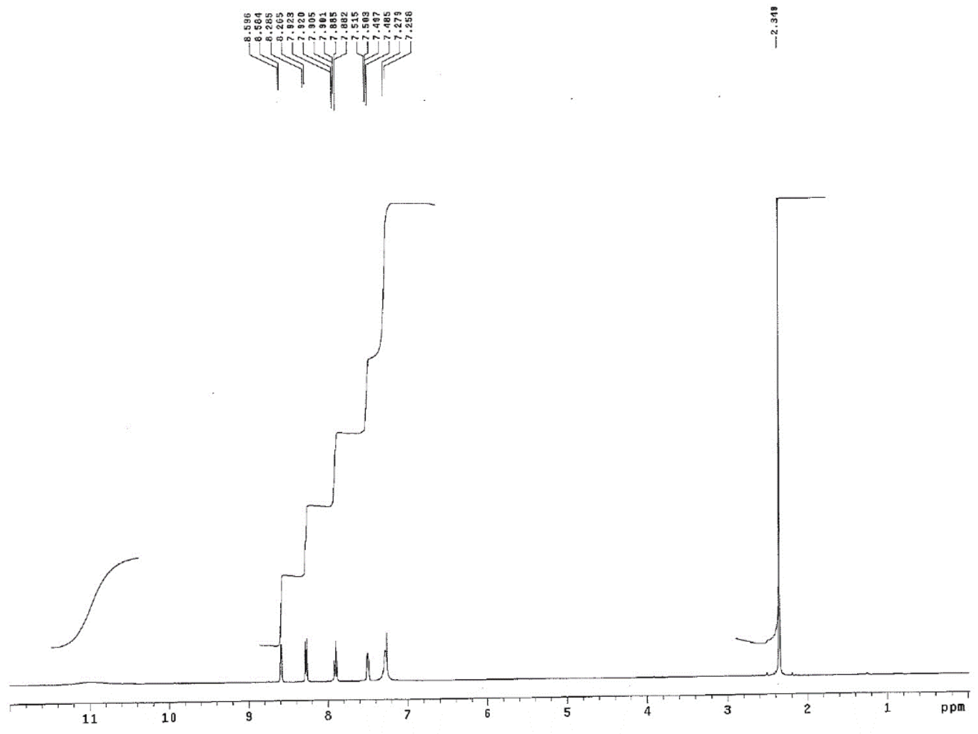

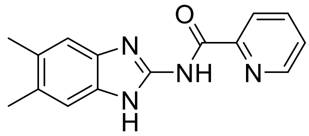

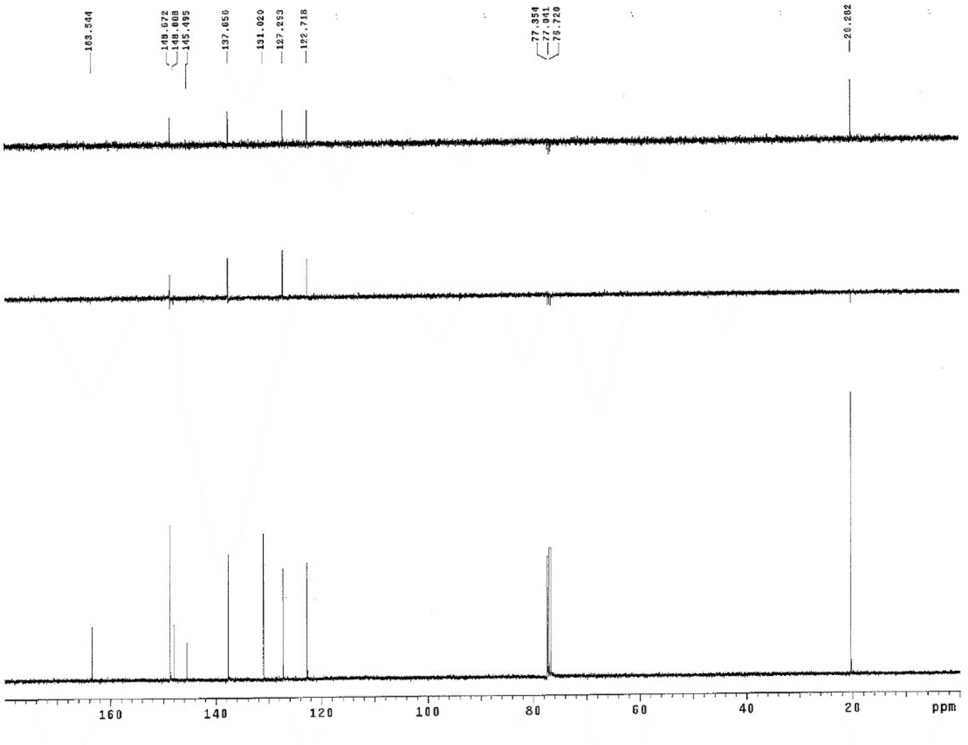

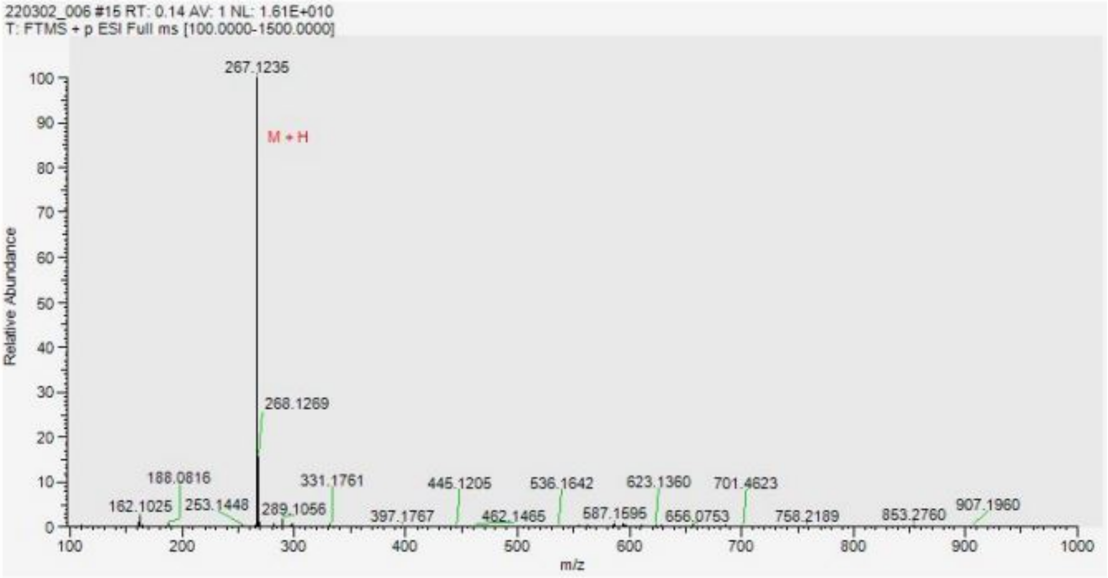
220302_006 #15 RT: 0.14 AV: 1 NL: 1.61E+010
T: FTMS + p ESI Full ms [100.0000-1500.0000]
267.1235
M + H
268.1269
188.0816
162.1025
253.1448
289.1056
331.1761
397.1767
445.1205
462.1465
536.1642
587.1595
623.1360
656.0753
701.4623
758.2189
853.2760
907.1960
Relative Abundance
m/z
